# Dr. William S. Chipley

**Published:** 1854-03

**Authors:** 


### DR. WILLIAM S. CHIPLEY.

WE are glad to learn that our friend, Dr. W. S. CHIPLEY, has been elected Professor of Theory and Practice of Medicine in Transylvania University. It is the best appointment the Trustees could have made. Dr. CHIPLEY is a man of real ability—a scholar, a student, a practitioner of large experience, a strong writer, and impressive, fluent lecturer. He has been one of our most esteemed correspondents, and was a short time since President of the Kentucky Medical Society.

